# Remediation of cobalt-polluted soil after application of selected substances and using oat (*Avena sativa* L.)

**DOI:** 10.1007/s11356-019-05052-x

**Published:** 2019-04-17

**Authors:** Milena Kosiorek, Mirosław Wyszkowski

**Affiliations:** Department of Environmental Chemistry, University of Warmia and Mazury in Olsztyn, Olsztyn, Poland

**Keywords:** Cobalt, Soil amendments, Trace elements, *Avena sativa* L

## Abstract

The aim of the study was to determine the effectiveness of soil application of manure, clay, charcoal, zeolite, and calcium oxide in remediation of soil polluted with cobalt (0, 20, 40, 80, 160, 320 mg Co kg^−1^ of soil). The following were determined: weight of harvested plants as well as the content of cobalt in grain, straw, and roots of oat. In addition, tolerance index (Ti), cobalt bioconcentration (BCF), translocation (TF), and transfer (TFr) coefficients were derived. In the series without amendments, the increasing doses of cobalt had a significant effect by decreasing the yields of oat grain and straw and the mass of its roots. Also, lower tolerance index values were noted in the objects polluted with cobalt, especially with its highest dose. The application of manure had the strongest effect on increasing the mass of particular organs of the test plant, while the application of charcoal led to a significant decrease in this respect. The application of all substances to the soil, and especially manure and calcium oxide, resulted in higher tolerance index Ti values. The growing contamination of soil with cobalt caused a significant increase in the content of this element in oat and in the values of the translocation coefficient, in contrast to the effects noted with respect to the bioconcentration and transfer coefficients. All the substances applied to soil reduced the content of cobalt and its bioconcentration in oat straw, in opposition to grain and roots, limited its translocation, but elevated the transfer of this element from soil to plants. Soil contamination with cobalt promoted the accumulation of lead and copper in grain, cadmium, lead, nickel, zinc, manganese, and iron in straw, as well as cadmium, nickel, zinc, and manganese in oat roots. As the cobalt dose increased, the content of other trace elements in oat organs either decreased or did not show any unambiguous changes. Of all the tested substances, the strongest influence on the content of trace elements was produced by calcium oxide in straw and roots and by zeolite in roots, whereas the weakest effect was generated by manure in oat grain. Oat is not the best plant for phytoremediation of soils contaminated with cobalt.

## Introduction

For their proper growth and development, plants need water, adequate temperature, and availability of nutrients in the soil. Adequate levels of nutrients in plants are difficult to maintain, mostly because of the simultaneous demand for macro- (nitrogen, phosphorus, calcium, magnesium, and sulphur) and micronutrients (cobalt, iron, copper, manganese, zinc, molybdenum, boron, and others) (Rinkins and Hollendorf 1982). The plant demand for cobalt is low. An average concentration of this element in plant tissues ranges from 0.03 to 0.55 mg kg^−1^ of dry matter (Sillanpää and Jansson 1992). The transfer coefficient for cobalt, which shows the ratio of cobalt concentration in plants to its content in soil, is 0.01–0.03 (Mascanzoni 1989). Its transport from roots to shoots and leaves is small. Plants absorb cobalt from soil most often in the form of divalent cobalt ions (International Plant Nutrition Institute 2015).

When the concentration of cobalt in plants is maintained on an adequate level, plants are able to produce cobalamin, essential for the production of vitamin B_12_, owing to which oxygen molecules can be bound. Cobalt is also responsible for limiting root infections and initiating the process of nodule formation (Yadov and Khanna 2002). When excessive levels of cobalt accumulate, the element can interfere with the course of chlorophyll synthesis by blocking iron transport to protoporphyrin, which affects the production of chlorophyll pigments (El-Sheekh et al. 2003). Excessive concentrations of cobalt in plants may also contribute to retardation of the growth of roots and shoots, which in turn can disturb the uptake of water and nutrients by plants. Consequently, both the quantity and quality of yields are significantly depressed (Hemantaranjan et al. 2000).

It is possible to control adverse effects of soil contamination with metals on the growth and development of plants, for example through soil remediation processes (Mulligan et al. 2001). Organic fertilisation, which is an example of soil remediation measures, improves the soil content of nutrients and, even more importantly, its content of organic matter (Stefanescu 2004). Soil amendments which are rich in clay particles significantly increase the sorption capacity of soil and improve its physical characteristics (Delgado and Gómez 2016). Immobilisation of trace elements in the soil can also be achieved by soil enrichment with substances that are potent adsorbents, e.g. zeolites (Eprikashvili et al. 2016). On the other hand, soil liming improves crop yields and stimulates the sorption of trace elements by the soil sorption concentration, as a result of which the treatment diminishes the mobility of these elements (Brodowska 2003; Gál et al. 2008).

A special role in the removal of trace elements from soil is played by phytoremediation treatments (Ali et al. 2013). Phytoremediation technologies involve plants as well as soil microorganisms closely associated with plants (Zaborowska et al. 2016). Owing to plant hyperaccumulators, it is possible to identify the mechanism of plant uptake of a given element from soil and to indicate where it accumulates the most within a plant organism (Ali et al. 2013). Phytoremediation treatments are claimed to be cost-effective and environmentally friendly solutions (Garbisu and Alkorta 2003).

The above considerations substantiate our research, whose aim was to determine the effectiveness of soil application of manure, clay, charcoal, zeolite, and calcium oxide in remediation of soil polluted with cobalt. The following were determined: weight of harvested plants as well as the content of cobalt and other trace elements in grain, straw, and roots of oat, which is a popular crop in Poland. In addition, tolerance index (Ti), cobalt bioconcentration (BCF), translocation (TF), and transfer (TFr) coefficients were derived.

## Materials and methods

### Methodological approach

A greenhouse pot experiment was conducted at the University of Warmia and Mazury in Olsztyn (north-eastern Poland). Polyethylene pots were each filled with 9 kg of the soil of the textural composition typical of loamy sand, and with the following percentages of the fractions: sand (> 0.05 mm)—73.9%, silt (0.002–0.05 mm)—24.1%, and clay (< 0.002 mm)—2.0%. The soil had the following parameters: reaction in 1 M KCl 5.05, hydrolytic acidity 28.40 mmol_(+)_ kg^−1^, total exchangeable bases 46.50 mmol_(+)_ kg^−1^, cation exchange capacity 74.90 mmol_(+)_ kg^−1^, base saturation 68.08%, and organic carbon content 12.15 g kg^−1^. The experiment was conducted in 6 series with 3 replicates. Series 1 consisted of soil without amendments, 2—with manure (granulated bovine manure), 3—with clay, 4—with charcoal, 5—with zeolite (clinoptilolite), and 6—with calcium oxide (50%). The soil amendments in series 2 to 5 were added to soil in amounts equal 2% of the soil mass in a pot, and calcium oxide was applied in a dose corresponding to 1 hydrolytic activity. At the onset of the experiment, soil was polluted once with one of the following doses of cobalt (CoCl_2_): 0, 20, 40, 80, 160, 320 mg kg^−1^ of soil, and fertilised with 100 mg N (NH_4_NO_3_), 35 mg P (KH_2_PO_4_), 100 mg K (KCl), 50 mg Mg (MgSO_4_ · 7H_2_O), 0.33 mg B (H_3_BO_3_), 5 mg Mn (MnCl_2_ · 4H_2_O) and 5 mg Mo ((NH_4_)_6_Mo_7_O_24_ · 4H_2_O) kg^−1^ of soil. The choice of the cobalt doses to be tested in the experiment was dictated by the allowable quantities of this element in different types of soil specified in the Regulation of the Minister of the Environment of 9 September 2002, on soil quality standards and earth quality standards, and in the currently binding Regulation of the Minister of the Environment of 1 September 2016 on conducting assessment of the contamination of the earth’s surface. Having prepared the soil, oat (*Avena sativa* L.) of the variety Zuch was sown in each pot. The experiment was performed with 15 plants per pot, and the soil moisture was kept at 60% of water capillary capacity. After 78 days, at full ripeness, oat was harvested for grain, straw, and roots.

### Methods of laboratory and statistical analyses

The collected plant material was weighed to determine the weight of individual plant organs. Prior to the determination of the content of trace elements, the plant material was dried at 60 °C, after which it was ground and wet mineralised in concentrated nitric acid (HNO_3_ of analytic purity grade) of the concentration of 1.40 g cm^−3^. The digestion was carried out in a MARS 5 microwave oven, with the plant material placed in HP500 Teflon® vessels.

Next, the content of cobalt, cadmium, lead, chromium, nickel, zinc, copper, manganese, and iron was determined with flame atomic absorption spectrometry (FAAS) in an acetylene-air flame (Ostrowska et al. 1991). The results were compared with the certified reference material NCS ZC 73030 from the China National Analysis Center for Iron & Steel 2014 and with reference solutions by Fluka assigned the symbols: Pb 16595, Cd 51994, Cr 02733, Ni 42242, Mn 63534, Zn 18827, Cu 38996, Fe 16596, and Co 119785.0100. Moreover, the following coefficients were calculated for cobalt determined in oat grain, straw, and roots: bioconcentration coefficient from the formula: (BCF) = C_plant organ_/C_soil_, translocation coefficient (TF) = C_aerial parts_/C_roots_, and transfer coefficient (TFr) = C_plant_/C_soil_, where C stands for cobalt content expressed in mg kg^−1^ (Ali et al. 2013; Mleczek et al. 2013). Moreover, tolerance index Ti was computed from the formula: Ti = biomass yield from a cobalt-polluted object / biomass yield from the control object. The tolerance index above 1 indicates a positive influence while Ti below 1 suggests a negative influence of cobalt on the growth and development of plants.

The soil analytical methods applied prior to the experiment were described by Wyszkowski and Sivitskaya (2013). The research results were processed statistically with a two-factor analysis of variance (ANOVA) supported by Statistica software (Dell Inc. 2016). Principal component analysis (PCA) and correlation coefficients served to evaluate the influence of cobalt and soil amendments on the content of trace elements in oat.

## Results

The research results suggest that the incorporation of increasing cobalt doses with the simultaneous addition of soil remediating substances such as manure, clay, charcoal, zeolite, and calcium oxide affected the plant biomass, tolerance index, and content of trace elements as well as cobalt bioconcentration, translocation, and transfer coefficients in oat grain, straw, and roots.

**Fig. 1:**
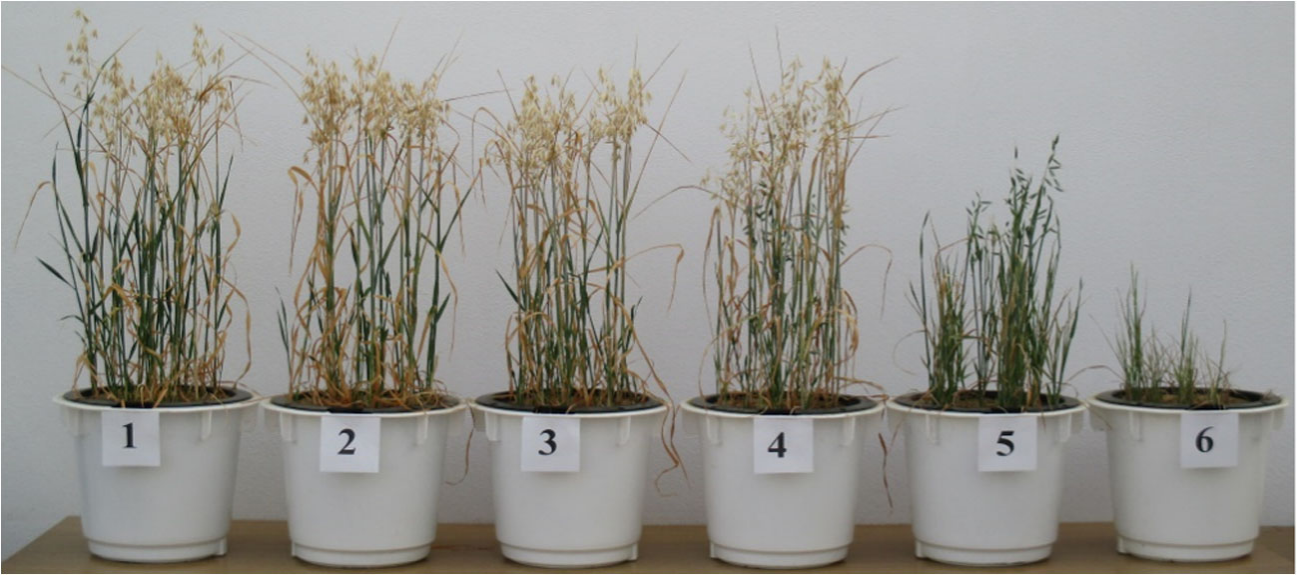
The effect of increasing doses of cobalt in the without amendments series on the oats. Explanations: (1) 0 mg, (2) 20 mg, (3) 40 mg, (4) 80 mg, (5) 160 mg, (6) 320 mg Co kg^−1^ of soil

**Fig. 2:**
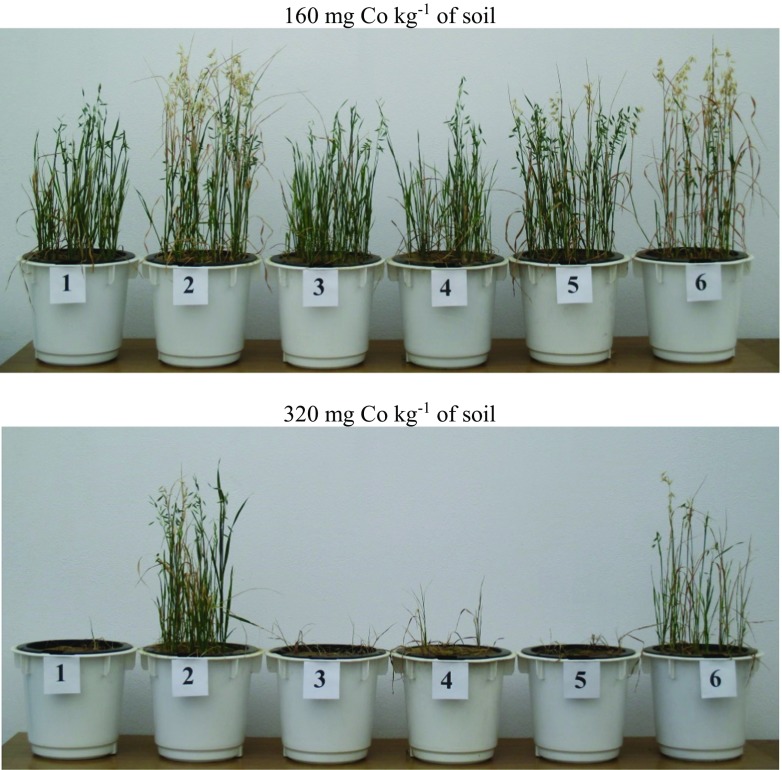
The effect of 160 and 320 mg Co kg ^−1^ of soil on the oats. Explanations: (1) without amendments, (2) manure, (3) clay, (4) charcoal, (5) zeolite, (6) calcium oxide

In the series without soil amendments, the applied cobalt doses caused a decrease in the mass of grain, straw, and roots of oat (Table 1). The strongest influence on grain (93%) was produced by the dose of 160 mg Co kg^−1^ of soil, while 320 mg Co kg^−1^ of soil had the most powerful effect on straw (95%) and roots (73%). These effects are best illustrated in Fig. 1, where it is evident that the yield of oat was much lower in pots polluted with these two doses of cobalt than in the other pots. However, it should be mentioned that oat plants were unable to form grain when exposed to the dose of 320 mg Co kg^−1^ of soil. After manure had been added to soil, a significant increase in straw yield (by 48% on average) and root mass (by 26%) was noted. The beneficial influence of manure and calcium oxide on the growth of the test plant is best illustrated in Fig. 2, which shows the highest oat plant growth in pots polluted with 160 and 320 mg Co kg^−1^ of soil and amended with these two substances. The other soil amendments did not have a positive effect on oat yields. The soil incorporation of charcoal had the strongest limiting effect on yields of grain (by 41% on average), straw (26%), and root mass (28%) of the test plant.

**Table 1:** Oats yield (g f. w. pot^−1^)

Dose of cobalt (mg kg^−1^ of soil)	Without amendments	Kind of substance neutralising effect of cobalt					
		Manure	Clay	Charcoal	Zeolite	Calcium oxide	Average
Grain							
0	16.00	13.61	10.69	9.28	9.95	9.63	11.53
20	14.92	12.07	9.64	8.65	9.09	9.09	10.58
40	13.89	11.95	9.79	8.25	9.15	9.01	10.34
80	12.38	10.58	7.90	6.13	9.77	10.34	9.52
160	1.07	8.98	1.80	2.05	2.63	7.04	3.93
320	0.00	2.64	0.00	0.00	0.00	0.71	0.56
Average	9.71	9.97	6.64	5.73	6.77	7.64	7.74
*r*	− 0.923**	− 0.992**	− 0.950**	− 0.963**	− 0.942**	− 0.938**	− 0.977**
LSD	a—1.10**, b—1.10**, a·b—2.70**						
Straw							
0	43.59	44.94	35.59	27.40	34.43	32.35	36.38
20	33.33	44.06	29.55	27.54	34.48	27.92	32.81
40	30.63	43.59	23.93	25.74	26.34	29.61	29.97
80	34.61	43.73	28.93	20.84	30.26	33.16	31.92
160	22.05	39.65	25.60	18.18	26.72	22.67	25.81
320	2.00	30.00	1.07	2.36	0.39	9.66	7.58
Average	27.70	40.99	24.11	20.35	25.44	25.89	27.41
*r*	− 0.966**	− 0.979**	− 0.923**	− 0.989**	− 0.938**	− 0.935**	− 0.972**
LSD	a—4.73**, b—4.73**, a·b—11.59**						
Roots							
0	3.31	3.79	2.82	2.12	2.44	2.39	2.81
20	2.85	3.38	2.27	2.34	2.64	2.28	2.63
40	2.62	2.84	1.94	2.19	2.08	2.11	2.30
80	3.17	3.15	2.69	1.82	2.63	2.30	2.63
160	3.07	3.54	3.26	2.37	4.18	1.93	3.06
320	0.91	3.44	0.32	0.65	0.19	1.88	1.23
Average	2.65	3.36	2.22	1.92	2.36	2.15	2.44
*r*	− 0.839**	0.098	− 0.662**	− 0.818**	− 0.501*	− 0.853**	− 0.713**
LSD	a—0.46**, b—0.46**, a·b—1.12**						

LSD for: a—cobalt dose, b—kind of neutralising substance, a·b—interaction

Significant at **P = 0.01, *P = 0.05; *r*—correlation coefficient

**Fig. 3:**
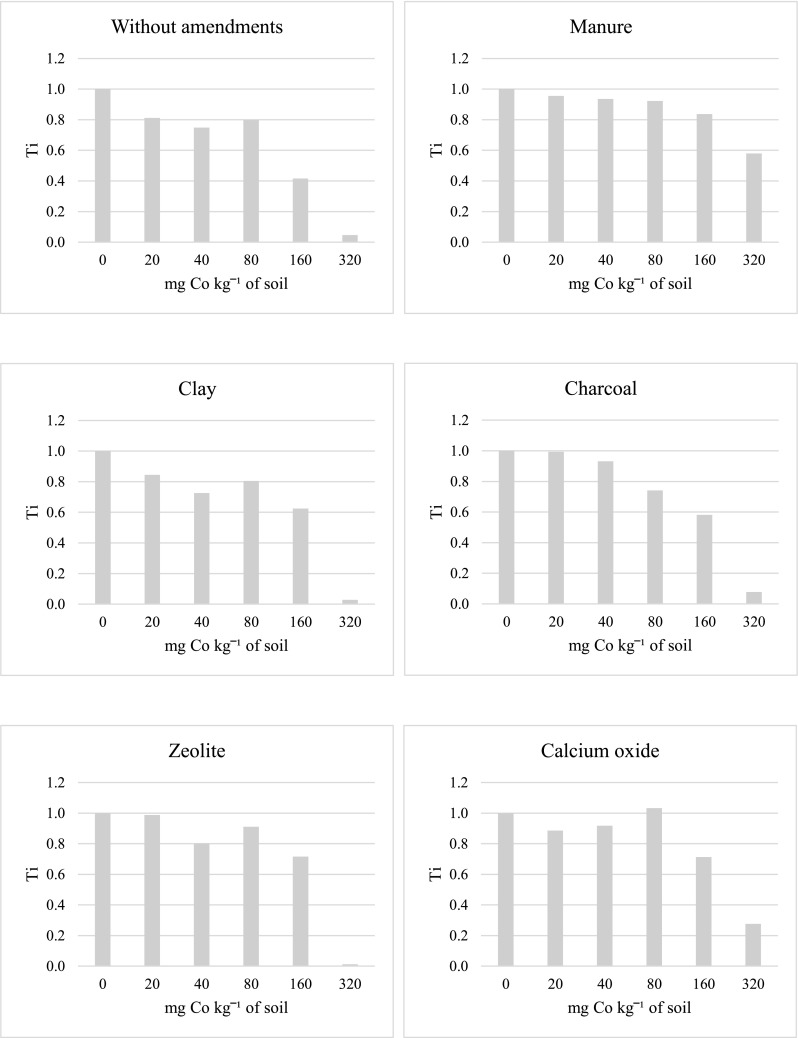
Tolerance index (Ti) for oats

In the series without soil amendments, soil contamination with cobalt lowered the tolerance index Ti from 1.000 to 0.046 (Fig. 3). All the soil amendments raised the Ti value for oat, with calcium oxide and manure having a stronger influence than zeolite, charcoal, and clay. The tolerance index Ti less than one indicates plant growth retardation, an effect which in our study occurred most often in the series without soil amendments. Unfortunately, despite soil amending substances, Ti values slightly higher than 1 were noted in only one cobalt-polluted object, i.e. in the series with calcium oxide.

In the series without soil amendments, in response to the increasing soil pollution with cobalt, the content of this element in oat grain, straw, and roots increased by 66, 311, and 145%, respectively (Table 2). All the applied substances had a positive effect by limiting the amount of cobalt, but only in oat straw. The decrease in the straw content of cobalt ranged on average from 28% in the series with clay to 57% in the soil amended with calcium oxide, compared to the series without amendments. Reverse relationships were noted in oat grain and roots. The strongest impact on the elevated cobalt content in grain was produced by clay (42%) and in roots—by zeolite (323%).

**Table 2:** Content of cobalt in oats grain, straw, and roots (mg kg^−1^ d. m)

Dose of cobalt (mg kg^−1^ of soil)	Without amendments	Kind of substance neutralising effect of cobalt					
		Manure	Clay	Charcoal	Zeolite	Calcium oxide	Average
Grain							
0	0.119	0.136	0.197	0.207	0.173	0.175	0.168
20	0.148	0.155	0.218	0.221	0.190	0.181	0.186
40	0.154	0.190	0.232	0.215	0.176	0.187	0.192
80	0.220	0.195	0.283	0.236	0.205	0.181	0.220
160	0.198	0.251	0.265	0.303	0.226	0.203	0.241
320	n. a.	0.248	n. a.	n. a.	n. a.	0.263	0.256
Average	0.168	0.196	0.239	0.236	0.194	0.198	0.210
*r*	0.784**	0.863**	0.791**	0.966**	0.937**	0.967**	0.921**
LSD	a—0.018**, b—0.018**, a·b—0.044**						
Straw							
0	0.096	0.103	0.106	0.024	0.063	0.066	0.076
20	0.101	0.108	0.106	0.076	0.072	0.083	0.091
40	0.140	0.116	0.124	0.091	0.067	0.082	0.103
80	0.193	0.125	0.107	0.102	0.085	0.086	0.116
160	0.313	0.157	0.162	0.139	0.145	0.092	0.168
320	0.395	0.239	0.280	0.235	0.214	0.125	0.248
Average	0.206	0.141	0.148	0.111	0.108	0.089	0.134
*r*	0.973**	0.993**	0.965**	0.975**	0.990**	0.962**	0.998**
LSD	a—0.045**, b—0.045**, a·b—0.110**						
Roots							
0	0.627	0.459	0.423	0.262	0.262	0.393	0.404
20	0.625	0.501	1.606	0.959	1.201	0.451	0.891
40	0.713	2.718	2.880	1.673	2.455	0.819	1.876
80	0.811	3.287	4.872	4.830	5.279	1.971	3.508
160	0.870	3.290	4.240	5.197	5.957	2.289	3.641
320	1.535	3.478	4.535	5.260	6.773	2.577	4.026
Average	0.864	2.289	3.093	3.030	3.655	1.417	2.391
*r*	0.973**	0.703**	0.698**	0.798**	0.860**	0.873**	0.820**
LSD	a—0.169**, b—0.169**, a·b—0.428**						

LSD for: a—cobalt dose, b—kind of neutralising substance, a·b—interaction

Significant at **P = 0.01, *P = 0.05; *r*—correlation coefficient; n.a.—not analysed

**Fig. 4:**
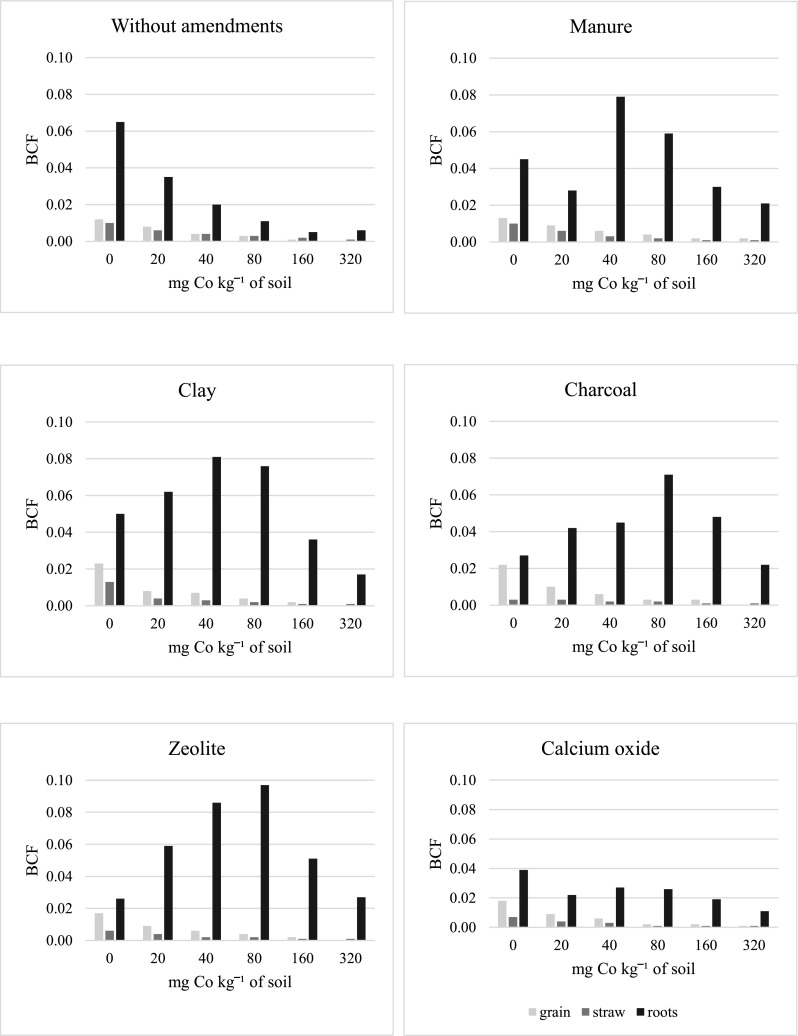
Bioconcentration factor (BCF) of cobalt in grain, straw, and roots oats

**Fig. 5:**
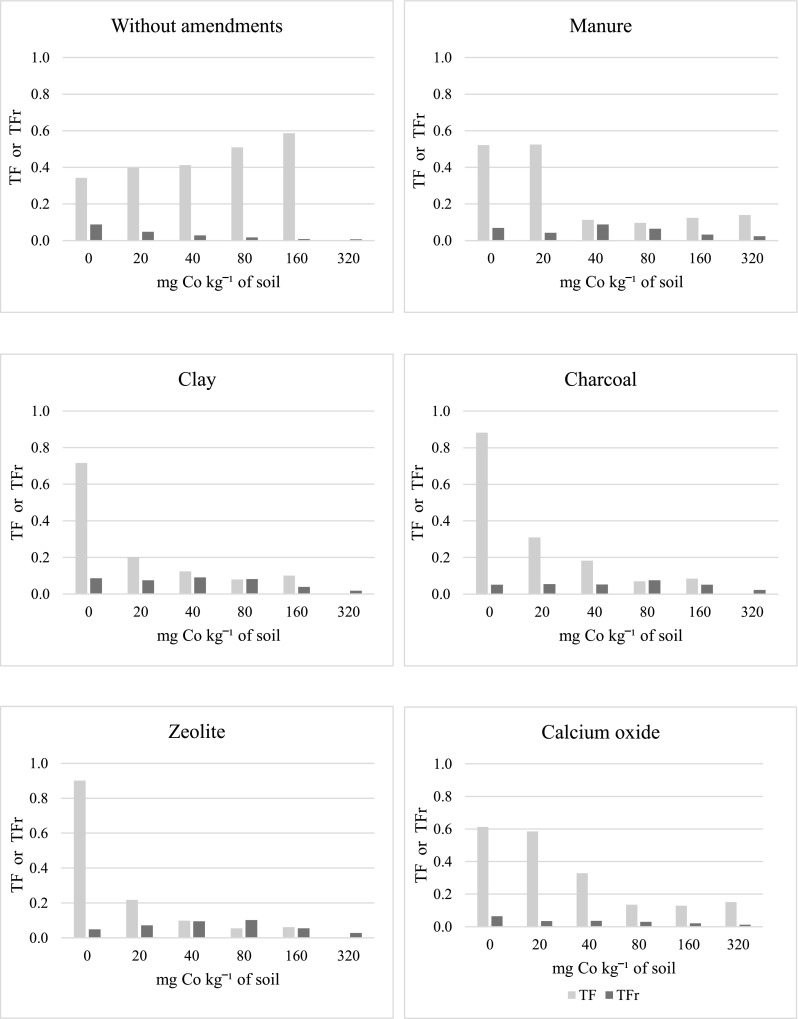
Translocation factor (TF) and transfer factor (TFr) of cobalt in oats

In the series without amendments, the increasing doses of cobalt significantly lowered the value of bioaccumulation factor (BCF) for cobalt in grain (by 12 times), straw (by 10 times), and roots of oat (by 11 times) (Fig. 4). Clay, charcoal, and zeolite contributed to the increase in the BFC values in grain, while manure, clay, charcoal, and zeolite had the same effect in oat roots. The strongest effect on BFC in grain was produced by clay and charcoal (risen by 50%), and in roots—by zeolite (risen by 142%). Charcoal caused the greatest reduction in the value of this factor in oat straw. In the series without soil amendments, soil contamination with cobalt significantly contributed to an increase in values of the translocation factor (TF) of cobalt (by 72%) while decreasing the transfer (TFr) of this element (by 72%) (Fig. 5). Soil amendment with any of the tested substances led to lower TR values, and most of these substances caused an increase in the cobalt TFr values. Application of clay, manure, and zeolite had the strongest impact with regard to the diminished cobalt TR from oat roots to aerial organs. Out of the tested substances, clay and zeolite demonstrated the strongest influence with respect to increasing the cobalt TFr values.

In the control series, after the application of 80 mg Co kg^−1^ of soil, the content of cadmium increased the highest in grain (100%), and when a dose of 320 mg Co kg^−1^ of soil was applied, the highest increase in this element appeared in straw (106%) and roots of oats (131%) (Table 3). The soil amendments reduced the cadmium content in grain (except CaO) and roots, while clay also lowered the straw concentration of this element. Charcoal and zeolite had the strongest limiting effect on Cd in oat grain (− 50% and − 47%, respectively), while manure was most effective in roots (− 26%). Reverse tendencies in changes in the cadmium content were observed in oat straw after application of charcoal, zeolite, and calcium oxide to soil. The highest increase in the Cd content of grain (38%) was noted after the soil had been treated with calcium oxide.

**Table 3:** Content of cadmium in oats grain, straw, and roots (mg kg^−1^ d. m)

Dose of cobalt (mg kg^−1^ of soil)	Without amendments	Kind of substance neutralising effect of cobalt					
		Manure	Clay	Charcoal	Zeolite	Calcium oxide	Average
Grain							
0	0.019	0.021	0.018	0.013	0.012	0.018	0.017
20	0.026	0.025	0.020	0.014	0.012	0.031	0.021
40	0.028	0.026	0.025	0.014	0.015	0.034	0.024
80	0.038	0.028	0.025	0.015	0.018	0.035	0.026
160	0.037	0.034	0.027	0.020	0.025	0.035	0.030
320	n. a.	0.037	n. a.	n. a.	n. a.	0.041	0.039
Average	0.030	0.029	0.023	0.015	0.016	0.032	0.026
*r*	0.869**	0.944**	0.854**	0.968**	0.990**	0.738**	0.973**
LSD	a—0.002**, b—0.002**, a·b—0.006**						
Straw							
0	0.032	0.043	0.029	0.064	0.041	0.056	0.044
20	0.040	0.043	0.032	0.057	0.064	0.055	0.049
40	0.040	0.047	0.045	0.064	0.054	0.064	0.052
80	0.049	0.052	0.050	0.067	0.057	0.066	0.057
160	0.061	0.048	0.049	0.066	0.070	0.076	0.062
320	0.066	0.061	0.053	0.064	0.071	0.078	0.066
Average	0.048	0.049	0.043	0.064	0.060	0.066	0.055
*r*	0.934**	0.917**	0.760**	0.332	0.753**	0.892**	0.914**
LSD	a—0.005**, b—0.005**, a·b—0.012**						
Roots							
0	0.065	0.071	0.061	0.077	0.082	0.086	0.074
20	0.085	0.072	0.088	0.078	0.080	0.085	0.081
40	0.071	0.082	0.085	0.083	0.080	0.086	0.081
80	0.088	0.084	0.089	0.082	0.089	0.091	0.087
160	0.146	0.072	0.088	0.083	0.081	0.098	0.095
320	0.150	0.069	0.086	0.087	0.092	0.088	0.095
Average	0.101	0.075	0.083	0.082	0.084	0.089	0.086
*r*	0.906**	− 0.423	0.378	0.877**	0.717**	0.352	0.871**
LSD	a—0.019**, b—0.019**, a·b—0.046**						

LSD for: a—cobalt dose, b—kind of neutralising substance, a·b—interaction

Significant at **P = 0.01, *P = 0.05; *r*—correlation coefficient; n.a.—not analysed

In soil without amendments, the increasing soil contamination with cobalt raised the lead content in grain (by 106%) and straw (by 1176%) relative to the control (without cobalt pollution) (Table 4). All the tested soil remediating substances limited the content of lead in oat grain, in addition to which manure caused the same result in oat straw. Noteworthy is a particularly strong effect of charcoal, clay, and calcium oxide, which reduced the lead content in oat grain by 38, 40, and 64%, respectively, compared to the series without amendments. Most of the applied substances had an opposite influence on the content of lead in oat straw and roots, with calcium oxide producing the strongest impact. This substance was conducive to an increase in the lead content in straw (39%) and roots of this crop (28%).

**Table 4:** Content of lead in oats grain, straw, and roots (mg kg^−1^ d. m)

Dose of cobalt (mg kg^−1^ of soil)	Without amendments	Kind of substance neutralising effect of cobalt					
		Manure	Clay	Charcoal	Zeolite	Calcium oxide	Average
Grain							
0	0.447	0.383	0.106	0.449	0.668	0.220	0.379
20	0.664	0.462	0.334	0.495	0.496	0.451	0.483
40	0.668	0.463	0.462	0.526	0.453	0.220	0.465
80	0.548	0.546	0.510	0.292	0.517	0.175	0.431
160	0.921	0.699	0.530	0.253	0.545	0.149	0.516
320	n. a.	0.544	n. a.	n. a.	n. a.	0.169	0.356
Average	0.650	0.516	0.388	0.403	0.536	0.231	0.439
*r*	0.811**	0.574*	0.772**	− 0.839**	− 0.202	− 0.496	− 0.337
LSD	a—0.254**, b—0.254**, a·b—0.622**						
Straw							
0	0.025	0.129	0.182	0.190	0.214	0.232	0.162
20	0.166	0.158	0.189	0.209	0.219	0.232	0.196
40	0.183	0.160	0.195	0.196	0.235	0.230	0.200
80	0.172	0.174	0.208	0.194	0.214	0.239	0.200
160	0.163	0.165	0.188	0.206	0.212	0.245	0.197
320	0.319	0.144	0.179	0.218	0.228	0.249	0.223
Average	0.171	0.155	0.190	0.202	0.220	0.238	0.196
*r*	0.827**	− 0.015	− 0.375	0.765**	0.215	0.934**	0.769**
LSD	a–0.019**, b–0.019**, a·b–0.045**						
Roots							
0	0.251	0.237	0.254	0.275	0.297	0.298	0.269
20	0.245	0.256	0.252	0.294	0.284	0.302	0.272
40	0.236	0.236	0.257	0.311	0.297	0.327	0.277
80	0.252	0.264	0.274	0.300	0.297	0.335	0.287
160	0.265	0.254	0.286	0.296	0.297	0.331	0.288
320	0.248	0.232	0.292	0.299	0.322	0.328	0.287
Average	0.250	0.247	0.269	0.296	0.299	0.320	0.280
*r*	0.293	− 0.306	0.916**	0.268	0.882**	0.562*	0.738**
LSD	a—0.015**, b—0.015**, a·b—0.037**						

LSD for: a—cobalt dose, b—kind of neutralising substance, a·b—interaction

Significant at **P = 0.01, *P = 0.05; *r*—correlation coefficient; n.a.—not analysed

In the pots untreated with any amendments, the soil contamination with cobalt had a relatively weak effect on the content of chromium in oat straw and roots, while being conducive to reducing the concentration of this element in grain (Table 5). Changes in the content of chromium in oat straw and roots were small (a few per cent), except for manure, which decreased the content of this element by 13%, and calcium oxide, which raised it by 15% compared to the series without amendments. Application of most of the substances to soil favoured the accumulation of chromium in oat grain, although its content was extremely low.

**Table 5:** Content of chromium in oats grain, straw, and roots (mg kg^−1^ d. m)

Dose of cobalt (mg kg^−1^ of soil)	Without amendments	Kind of substance neutralising effect of cobalt					
		Manure	Clay	Charcoal	Zeolite	Calcium oxide	Average
Grain							
0	0.004	0.001	0.002	0.003	0.004	0.005	0.003
20	0.002	0.001	0.003	0.004	0.005	0.005	0.003
40	0.002	0.002	0.003	0.004	0.005	0.005	0.004
80	0.001	0.001	0.003	0.004	0.005	0.006	0.003
160	0.001	0.001	0.003	0.005	0.005	0.006	0.004
320	n. a.	0.001	n. a.	n. a.	n. a.	0.006	0.003
Average	0.002	0.001	0.003	0.004	0.005	0.006	0.003
*r*	− 0.866**	− 0.079	0.857**	0.840**	0.442	0.696**	0.241
LSD	a—0.0004**, b—0.0004**, a·b—0.001**						
Straw							
0	0.612	0.477	0.623	0.668	0.522	0.634	0.589
20	0.634	0.522	0.601	0.668	0.589	0.623	0.606
40	0.589	0.589	0.545	0.657	0.645	0.612	0.606
80	0.589	0.545	0.612	0.645	0.634	0.668	0.616
160	0.578	0.601	0.657	0.668	0.634	0.657	0.632
320	0.578	0.545	0.612	0.623	0.645	0.645	0.608
Average	0.597	0.546	0.608	0.655	0.612	0.640	0.610
*r*	− 0.688**	0.338	0.299	− 0.785**	0.583*	0.418	0.403
LSD	a—0.067**, b—0.067**, a·b—0.164**						
Roots							
0	0.645	0.545	0.556	0.645	0.634	0.645	0.612
20	0.679	0.612	0.634	0.690	0.657	0.701	0.662
40	0.589	0.455	0.668	0.668	0.713	0.746	0.640
80	0.645	0.601	0.679	0.724	0.814	0.746	0.701
160	0.668	0.601	0.589	0.735	0.735	0.870	0.700
320	0.668	0.589	0.724	0.690	0.657	0.757	0.681
Average	0.649	0.567	0.642	0.692	0.701	0.744	0.666
*r*	0.355	0.325	0.588*	0.387	− 0.011	0.529*	0.559*
LSD	a—0.062**, b—0.062**, a·b—0.153**						

LSD for: a—cobalt dose, b—kind of neutralising substance, a·b—interaction

Significant at **P = 0.01, *P = 0.05; *r*—correlation coefficient; n.a.—not analysed

In the series without soil amendments, the soil contamination with cobalt did not demonstrate an unambiguous effect on the content of nickel in oat straw or roots, although it depressed the accumulation of this element in grain (Table 6). However, the content of nickel in oat straw and roots harvested from the pots with the highest cobalt doses was higher than that in the control. All the substances reduced the content of nickel in oat aerial organs. In response to the application of these substances, a decrease in the nickel content in the aerial parts of oat plants ranged from 31% (charcoal) to 52% (clay) in grain, and from 25% (manure) to 82% (calcium oxide) in straw. None of the tested substances (except calcium oxide) had a significant influence with respect to an increase in the nickel content in oat roots (12%).

**Table 6:** Content of nickel in oats grain, straw, and roots (mg kg^−1^ d. m)

Dose of cobalt (mg kg^−1^ of soil)	Without amendments	Kind of substance neutralising effect of cobalt					
		Manure	Clay	Charcoal	Zeolite	Calcium oxide	Average
Grain							
0	1.259	0.543	0.404	0.530	0.681	0.425	0.640
20	1.337	0.443	0.386	0.547	0.660	0.425	0.633
40	0.269	0.295	0.412	0.781	0.438	0.490	0.448
80	0.721	0.334	0.360	0.495	0.356	0.486	0.459
160	0.469	0.560	0.373	0.460	0.408	0.456	0.454
320	n. a.	0.386	n. a.	n. a.	n. a.	0.404	0.395
Average	0.811	0.427	0.387	0.563	0.509	0.448	0.505
*r*	− 0.610*	− 0.062	− 0.627**	− 0.417	− 0.737**	− 0.399	− 0.741**
LSD	a—0.130**, b—0.130**, a·b—0.317**						
Straw							
0	0.456	0.365	0.382	0.095	0.074	0.130	0.250
20	0.464	0.391	0.373	0.087	0.061	0.078	0.242
40	0.356	0.286	0.308	0.087	0.139	0.056	0.205
80	0.477	0.339	0.260	0.126	0.065	0.061	0.221
160	0.512	0.347	0.269	0.126	0.135	0.091	0.247
320	0.521	0.360	0.334	0.143	0.243	0.091	0.282
Average	0.464	0.348	0.321	0.111	0.119	0.085	0.241
*r*	0.630**	0.069	− 0.278	0.873**	0.888**	− 0.003	0.687**
LSD	a—0.061**, b—0.061**, a·b—0.150**						
Roots							
0	0.473	0.608	0.313	0.412	0.313	0.417	0.422
20	0.547	0.651	0.438	0.703	0.273	0.486	0.517
40	0.347	0.556	0.286	0.326	0.286	0.538	0.390
80	0.378	0.295	0.412	0.230	0.629	0.547	0.415
160	0.642	0.317	0.681	0.521	0.681	0.595	0.573
320	0.534	0.408	0.825	0.725	0.547	0.694	0.622
Average	0.487	0.472	0.493	0.486	0.455	0.546	0.490
*r*	0.402	− 0.574*	0.935**	0.491	0.621*	0.944**	0.808**
LSD	a—0.071**, b—0.071**, a·b—0.173**						

LSD for: a—cobalt dose, b—kind of neutralising substance, a·b—interaction

Significant at **P = 0.01, *P = 0.05; *r*—correlation coefficient; n.a.—not analysed

In the control series, the impact of high cobalt doses on the zinc content was analogous to that exerted on the content of nickel in oat (Table 7). The highest cobalt dose led to a significant increase in the content of zinc in straw (53%) and roots (26%) of oat. A decrease in the grain content of zinc (16%) was observed in the soil polluted with 160 mg Co kg^−1^ of soil. Following the application of calcium oxide, the highest decline in the zinc content was noted in grain (9%), straw (30%), and roots (34%). All the other substances limited the content of zinc in roots, while manure and charcoal had the liming effect on zinc also in oat straw. Clay increased the content of zinc in oat roots, whereas charcoal, clay, and zeolite in oat grain.

**Table 7:** Content of zinc in oats grain, straw, and roots (mg kg^−1^ d. m)

Dose of cobalt (mg kg^−1^ of soil)	Without amendments	Kind of substance neutralising effect of cobalt					
		Manure	Clay	Charcoal	Zeolite	Calcium oxide	Average
Grain							
0	17.15	18.07	19.56	20.47	20.56	16.88	18.78
20	15.23	17.68	17.05	17.45	17.75	13.93	16.52
40	14.77	14.96	16.95	17.49	16.55	13.77	15.75
80	16.41	14.28	16.44	16.96	15.96	13.47	15.59
160	14.44	14.63	16.47	15.91	16.14	13.25	15.14
320	n. a.	14.54	n. a.	n. a.	n. a.	14.28	14.41
Average	15.60	15.69	17.29	17.66	17.39	14.26	16.03
*r*	− 0.558*	− 0.626**	− 0.669**	− 0.806**	− 0.709**	− 0.307	− 0.765**
LSD	a—0.555**, b—0.555**, a·b—1.359**						
Straw							
0	12.81	13.98	15.14	13.22	13.30	10.78	13.21
20	12.49	13.91	13.74	11.94	12.02	9.204	12.22
40	12.78	13.08	14.93	12.43	12.10	9.343	12.45
80	13.69	13.79	15.36	13.00	13.69	9.481	13.17
160	17.29	13.37	16.72	16.11	16.40	10.39	15.05
320	19.66	14.73	19.69	16.54	20.74	13.19	17.42
Average	14.79	13.81	15.93	13.87	14.71	10.40	13.92
*r*	0.974**	0.569*	0.962**	0.888**	0.972**	0.832**	0.963**
LSD	a—0.453**, b—0.453**, a·b—1.109**						
Roots							
0	24.46	20.16	17.97	16.29	19.62	15.36	18.98
20	22.44	21.50	18.04	17.73	20.00	15.29	19.17
40	20.14	20.92	19.80	15.85	21.94	12.93	18.60
80	23.74	18.80	21.24	19.67	22.92	14.90	20.21
160	24.71	19.96	24.61	24.66	23.01	18.50	22.57
320	30.89	22.27	31.25	22.69	26.89	20.34	25.72
Average	24.40	20.60	22.15	19.48	22.40	16.22	20.88
*r*	0.863**	0.432	0.998**	0.789**	0.957**	0.880**	0.985**
LSD	a—1.253**, b—1.253**, a·b—3.070**						

LSD for: a—cobalt dose, b—kind of neutralising substance, a·b—interaction

Significant at **P = 0.01, *P = 0.05; *r*—correlation coefficient; n.a.—not analysed

In the soil without amendments, an application of 80 mg Co kg^−1^ of soil resulted in an increase in the copper content in grain by 46% relative to the control (Table 8). The influence of cobalt on the content of this element in straw and roots was relatively weak, although the dose of 320 mg Co kg^−1^ of soil limited the accumulation of copper in oat straw by 18%. Most of the substances applied to soil induced the lowering of copper concentrations in all tested organs of oat. Compared to the series without amending substances, the decrease varied from 39% (clay) to 79% (calcium oxide) in grain, from 7–8% (clay, manure) to 20% (charcoal) in straw, and from 32% (manure) to 56–58% (zeolite, calcium oxide) in roots.

**Table 8:** Content of copper in oats grain, straw, and roots (mg kg^−1^ d. m)

Dose of cobalt (mg kg^−1^ of soil)	Without amendments	Kind of substance neutralising effect of cobalt					
		Manure	Clay	Charcoal	Zeolite	Calcium oxide	Average
Grain							
0	4.237	6.112	3.727	1.723	1.300	1.149	3.041
20	4.346	6.322	3.402	1.765	1.367	1.099	3.050
40	6.064	5.845	3.402	1.533	1.393	1.036	3.212
80	6.177	5.395	2.724	1.301	1.231	1.014	2.974
160	5.800	5.271	2.950	1.195	1.215	1.231	2.944
320	n. a.	4.397	n. a.	n. a.	n. a.	1.036	2.716
Average	5.325	5.557	3.241	1.504	1.301	1.094	2.990
*r*	0.639**	− 0.962**	− 0.773**	− 0.928**	− 0.719**	− 0.105	− 0.876**
LSD	a—0.207**, b—0.207**, a·b—0.506**						
Straw							
0	0.952	0.795	0.876	0.706	0.882	0.948	0.860
20	0.895	0.816	0.830	0.746	0.969	0.895	0.858
40	0.901	0.870	0.835	0.749	0.931	0.896	0.864
80	0.945	0.802	0.876	0.756	0.927	0.644	0.825
160	0.968	0.825	0.819	0.701	0.930	0.565	0.801
320	0.777	0.906	0.818	0.693	0.946	0.559	0.783
Average	0.906	0.836	0.842	0.725	0.931	0.751	0.832
*r*	− 0.687**	0.731**	− 0.579*	− 0.609*	0.298	− 0.839**	− 0.935**
LSD	a—0.056**, b—0.056**, a·b—0.137**						
Roots							
0	2.807	1.960	1.462	1.431	1.226	0.984	1.645
20	2.676	2.120	1.433	1.326	1.237	0.850	1.607
40	2.755	2.209	1.357	1.220	1.165	0.890	1.599
80	2.584	1.724	1.328	1.280	1.068	0.853	1.473
160	2.670	1.464	1.381	1.321	1.093	1.738	1.611
320	2.612	1.446	1.382	1.107	1.023	1.697	1.544
Average	2.684	1.821	1.390	1.281	1.135	1.169	1.580
*r*	− 0.603*	− 0.828**	− 0.333	− 0.770**	− 0.853**	0.844**	− 0.368
LSD	a—0.118**, b—0.118**, a·b—0.289**						

LSD for: a—cobalt dose, b—kind of neutralising substance, a·b—interaction

Significant at **P = 0.01, *P = 0.05; *r*—correlation coefficient; n.a.—not analysed

In the series without substances intended to alleviate cobalt contamination effects, the content of manganese significantly increased in oat straw, by the maximum of 92% after an application of 320 mg Co kg^−1^ of soil (Table 9). The influence of cobalt on the content of manganese in grain and roots was less equivocal. However, it is worth noticing that the content of this element in oat roots from the object contaminated with 320 mg Co kg^−1^ of soil was 67% higher than in the control. The application of each of the tested substances depressed the content of manganese in oat straw and most of them (except clay) in roots. Manure and, especially, calcium oxide had analogous effects on the manganese content in oat grain. After calcium oxide had been added to soil, the highest decrease in the manganese content in grain reached 40%, in straw—67%, and in roots—64%. A similar effect on the content of manganese in straw was generated by manure (− 55%). Contrary and relatively small effects on the content of manganese were produced by charcoal and zeolite in grain, and clay in roots.

**Table 9:** Content of manganese in oats grain, straw, and roots (mg kg^−1^ d. m)

Dose of cobalt (mg kg^−1^ of soil)	Without amendments	Kind of substance neutralising effect of cobalt					
		Manure	Clay	Charcoal	Zeolite	Calcium oxide	Average
Grain							
0	218.5	163.3	229.3	261.7	246.9	140.2	210.0
20	202.7	165.8	211.8	238.4	226.4	101.2	191.1
40	200.7	165.4	219.2	230.2	217.7	126.8	193.3
80	286.5	159.2	237.2	214.3	206.5	120.3	204.0
160	195.9	229.5	201.0	235.3	316.8	120.3	216.5
320	n. a.	198.0	n. a.	n. a.	n. a.	182.7	190.3
Average	220.9	180.2	219.7	236.0	242.9	131.9	200.9
*r*	0.010	0.656**	− 0.491	− 0.467	0.683**	0.754**	− 0.170
LSD	a—6.5**, b—6.5**, a·b—16.0**						
Straw							
0	486.8	249.7	401.9	465.3	456.1	219.6	379.9
20	496.2	250.3	459.3	437.9	453.4	116.1	368.9
40	498.3	263.7	453.2	426.5	468.0	178.5	381.4
80	502.3	252.2	439.2	422.5	517.8	163.3	382.9
160	667.5	269.1	548.1	461.2	566.5	188.1	450.1
320	934.5	312.3	666.8	399.2	865.1	326.5	584.1
Average	597.6	266.2	494.7	435.4	554.5	198.7	424.5
*r*	0.980**	0.950**	0.972**	− 0.587*	0.976**	0.792**	0.976**
LSD	a—8.9**, b—8.9**, a·b—21.8**						
Roots							
0	253.3	237.8	180.4	242.3	269.2	66.69	208.3
20	230.4	248.4	224.9	229.3	292.5	64.42	215.0
40	180.8	233.8	261.3	194.8	254.5	68.21	198.9
80	216.6	201.2	213.3	195.5	256.2	80.72	193.9
160	349.8	214.9	311.3	203.1	241.6	118.0	239.8
320	423.7	211.5	542.8	159.2	206.5	195.5	289.9
Average	275.7	224.6	289.0	204.0	253.4	98.9	224.3
*r*	0.898**	− 0.623**	0.961**	− 0.856**	− 0.918**	0.990**	0.918**
LSD	a—25.6**, b—25.6**, a·b—62.7**						

LSD for: a—cobalt dose, b—kind of neutralising substance, a·b—interaction

Significant at **P = 0.01, *P = 0.05; *r*—correlation coefficient; n.a.—not analysed

In the control series, an application of 320 mg Co kg^−1^ of soil effected the highest increase in the content of iron in straw (48%), while a dose of 160 mg Co kg^−1^ of soil resulted in a decrease in the grain content of iron (66%) (Table 10). The effect of cobalt on the content of iron in oat roots was unequivocal. Application of every tested substance to soil depressed the content of iron in the analysed oat organs. It needs to be underlined that charcoal caused the greatest decrease in the iron content in grain (51%), while calcium oxide had the most limiting influence on the iron content in straw (50%) and roots (57%) of oat.

**Table 10:** Content of iron in oats grain, straw, and roots (mg kg^−1^ d. m)

Dose of cobalt (mg kg^−1^ of soil)	Without amendments	Kind of substance neutralising effect of cobalt					
		Manure	Clay	Charcoal	Zeolite	Calcium oxide	Average
Grain							
0	44.45	15.68	14.73	13.62	14.37	15.69	19.76
20	42.02	15.27	15.11	13.01	13.69	15.11	19.03
40	15.98	13.16	13.40	13.87	13.20	14.55	14.03
80	18.51	14.11	12.93	13.63	11.76	15.47	14.40
160	15.34	16.18	13.20	12.08	15.46	13.00	14.21
320	n. a.	13.04	n. a.	n. a.	n. a.	13.13	13.09
Average	27.26	14.57	13.87	13.24	13.70	14.49	15.75
*r*	− 0.745**	− 0.393	− 0.716**	− 0.734**	0.307	− 0.825**	− 0.703**
LSD	a—0.82**, b—0.82**, a·b—2.00**						
Straw							
0	16.72	15.57	13.88	11.24	10.56	10.23	13.03
20	17.01	16.79	12.76	11.81	10.42	9.37	13.03
40	16.90	17.43	12.72	11.24	10.95	9.91	13.19
80	19.87	17.71	10.24	12.70	10.35	9.92	13.47
160	19.96	16.75	10.73	12.09	11.52	9.55	13.43
320	24.67	14.38	38.36	11.49	14.79	8.52	18.70
Average	19.19	16.44	16.45	11.77	11.43	9.58	14.14
*r*	0.973**	− 0.611*	0.826**	0.061	0.941**	− 0.854**	0.913**
LSD	a—1.48**, b—1.48**, a·b—3.62**						
Roots							
0	150.50	201.70	89.12	97.39	93.08	71.58	117.23
20	158.90	168.40	87.59	97.92	103.00	60.82	112.77
40	127.70	145.10	113.30	75.36	104.30	59.41	104.20
80	165.50	114.70	113.00	81.45	172.40	59.97	117.84
160	184.20	125.50	176.50	125.80	188.10	60.32	143.40
320	141.60	172.20	119.20	127.40	76.09	89.58	121.01
Average	154.73	154.60	116.45	100.89	122.83	66.95	119.41
*r*	0.032	− 0.019	0.490	0.751**	− 0.061	0.720**	0.442
LSD	a—16.65**, b—16.65**, a·b—40.78**						

LSD for: a—cobalt dose, b—kind of neutralising substance, a·b—interaction

Significant at **P = 0.01, *P = 0.05; *r*—correlation coefficient; n.a.—not analysed

## Discussion

Excessive soil content of trace elements, including cobalt, not only reduces soil fertility, but also disrupts physiological and biochemical functions of plants, thereby having a significant reducing impact on plant yields reduction (McGrath et al. 1995; El-Sheekh et al. 2003).

Limited rates and efficiency of plant photosynthesis and lower yields due to excessive accumulation of cobalt in plants are a consequence of the smaller assimilation surface of plants, damage to the photosynthetic apparatus, and a lower content of photosynthetic pigments (Liu et al. 2000; Ali et al. 2010). Relationships between high concentrations of cobalt in soil substrate and a decreasing content of chlorophyll in bean leaves were confirmed by Zengin and Munzuroglu (2005). Chaudhari et al. (2017) reported that a dose of 20 mg Co kg^−1^ of loam soil had the strongest influence on the growth of maize plants and the harvested fresh matter of this crop (71.38 cm after 30 days; 98.90 cm after 60 days; 222.73 g pot^−1^), while a dose of 80 mg Co kg^−1^ limited the growth of maize (61.18 cm after 30 days; 80.79 cm after 60 days, 166.03 g pot^−1^). Based on a 30-day pot experiment, Jayakumar et al. (2007) concluded that a dose of 50 mg Co kg^−1^ of soil had a stimulating effect on the growth of *Raphanus sativus*, although when doses of the pollutant increased up to 250 mg Co kg^−1^ of soil, the length of roots and shoots as well as the total leaf surface were significantly reduced. Strong impact of high cobalt doses, responsible to the lower plant height and yield, may be associated with the activity of catalase and peroxidase—enzymes belonging to oxidoreductases, whose activity increases parallel to the increasing contamination with cobalt, an event that eventually leads to the decomposition of products of photosynthesis (Kandil et al. 2013; Chaudhari et al. 2017). Positive influence of a small and permissible quantity of cobalt in soil (20 mg Co kg^−1^ of soil) confronted with the adverse effects of high cobalt doses (160 and 320 mg Co kg^−1^ of soil) on the plant height and harvested yields has been verified in the current study, which is best illustrated by the attached photographs (Figs. 1, 2, and 3) as well as data regarding oat mass (Table 1).

Furthermore, a negative effect of cobalt was also observed in the soil polluted with its highest dose, which prevented the formation of grain by oat plants, and consequently did not allow us to make laboratory analysis of grain from this treatment. The study performed by Stefanescu (2004) suggests that manure acts positively not only on soil fertility but also on wheat and maize yielding. According to Muthaura et al. (2017), insufficient concentrations of nitrogen and phosphorus in soil significantly decreased the number of leaves formed by maize plants, which translated into inferior grain yields. The above dependences explain the elevated yields of straw and roots of oats observed in our research after the soil had been amended with manure.

The research reported by Aery and Jagetiya (2000) indicates that the content of cobalt in wheat grown on sandy clay soil is much higher than in wheat cultivated on sandy soil, which has a significant effect on the volume of harvested yield. It is therefore plausible that soil enrichment with clay decreases the mobility of considerable amounts of cobalt, thereby improving the yields of the test crop. According to Kuzyakov et al. (2009), a positive effect on the growth of plants is also observed following soil amendment with charcoal. The study by Gujejiani et al. (2002) suggests that application of zeolites, characterised by a very high content of calcium, has a beneficial influence on the growth of plants and yield of grain as well as the yields of the other aerial organs of maize. As reported by Kovačević and Rastija (2010), liming treatments repeated four times on soil of the pH_KCl_ 3.74 and consisting of the following doses of dolomite with 56% content of CaO: 5, 10, and 15 t ha^−1^, resulted in a 15% increase in the maize yield harvested in the first year, 25%—in the second, 134%—in the third, and 50%—in the fourth year. The above relationships were not confirmed by our results, where the application of clay, charcoal, zeolite, and calcium oxide was followed by harvesting decreased oat yields. The application of compost and calcium oxide to polluted soil may have positive effects on yields of oat, maize (Wyszkowski and Ziółkowska 2009, 2011), and yellow lupine (Wyszkowski and Ziółkowska 2011).

The presence of high cobalt concentrations in soil is also conducive to certain disturbances in the plant uptake of other nutrients from the soil. The study by Jayakumar et al. (2007) demonstrated that an application of 50 mg cobalt kg^−1^ of soil had a substantial effect on the accumulation of the highest levels of copper, zinc, manganese, and iron in leaves of *Raphanus sativus*, whereas a dose of 250 mg cobalt kg^−1^ of soil resulted in the lowest concentrations of the same elements in leaves of the test plant. Gad (2006) showed a reverse tendency. When a dose of 8 mg Co kg^−1^ of soil had been added, starting with the control series and then treatments with urea, ammonium nitrate, and peanut compost, the content of cobalt increased in shoots of *Pisum sativum* L. (from 1.67, 3.55, 4.93, to 6.24 mg kg^−1^), similarly to that of manganese (from 27.1, 32.0, 36.0, to 45.9 mg kg^−1^) and zinc (from 10.6, 18.5, 22, to 29.3 mg kg^−1^).

In this study, the increasing doses of cobalt only effected a decrease in the content of zinc in oat grain, copper in straw and roots, and increased accumulation of zinc in straw and roots, copper in grain, and manganese in all test plant organs. Moreover, in the study by Gad (2006), antagonistic interaction was noted between cobalt and iron, as the increased cobalt content in the stem of the test plant was accompanied by a decrease in the iron content, which fell from 42.6 mg in the control series to 40.0 mg in the treatment with urea, 36.6 mg in the series with ammonium nitrate, and 34 mg kg^−1^ in objects treated with peanut compost. According to Palit et al. (1994), translocation of cobalt in higher plants is similar to transport of iron (active or passive), which explains the occurrence of antagonistic interactions, particularly between cobalt versus iron and manganese, and other elements present in the soil. This study proves the appearance of negative correlations between cobalt versus manganese and iron only in oat grain, where the content of the two latter elements decreased in plants exposed to the highest cobalt doses. Increased concentrations of manganese and iron were recorded in the other test plant organs, which disagrees with the experimental data reported by Palit et al. (1994). Research by Kabata-Pendias and Mukherjee (2007) prove that the presence of zinc in the soil solution may improve the availability of cadmium to plants. This relationship was also confirmed in our experiment, because the applied doses of cobalt caused an increase in the content of zinc and cadmium in oat straw and roots. The existence of synergistic relationships between cobalt versus cadmium and lead in *Brassica oleracea* has been demonstrated by Kalavrouziotis et al. (2009). According to Homer et al. (1991), antagonistic interactions may occur between cobalt and nickel, due to their competition for semi-selective transporter proteins present in roots of *Allyssum*. The research by Zeid (2001) shows possible interactions between cobalt and chromium which can affect the activity of amylase and transport of sugars to so-called embryonic axes.

Application of different substances to soil significantly affects the levels of trace elements in soil and their uptake by plants (Wyszkowski 2017). Bibak (1994) demonstrated that the uptake of cobalt by winter wheat grown on clay soil fertilised with bovine manure was higher than by wheat on the control unfertilised soil. The positive influence of bovine manure consisting of the elevated uptake of cobalt as well as zinc, copper, and iron by maize has been demonstrated by Ismail et al. (1996). Bhattacharyya et al. (2008) claimed that organic and mineral fertilisation differentiated the content of cobalt in rice straw and grain. They also proved the presence of dependences between the content of cobalt in rice shoots and the amounts of its forms dissolved in water, bound and exchangeable with iron and manganese (oxide form), or its lack, relative to cobalt bound in carbonates, with organic matter or its residual form. As reported by Kumpiene et al. (2008), a higher content of the organic substance in soil might reduce the uptake of trace elements by plants. This has also been proven in our study, where the content of cadmium in roots, lead in straw, and chromium in all organs of oat decreased. Finžgar et al. (2006) suggest that immobilisation of trace elements in soil may occur as a result of soil enrichment with amendments containing much of clay particles. In our experiment, when soil had been amended with clay, the content of only two elements (cadmium in straw and nickel in grain) decreased. A reverse effect was noted for cobalt in grain, zinc in straw, and manganese in roots. Incorporation of charcoal to soil might stimulate the uptake of macro- and micronutrients by plants (Van Zwieten et al. 2010), which was also verified in the current study, especially with respect to the content of zinc in grain and chromium in straw of oat. In the study by Ciećko et al. (2001), application of lignite, compost, and calcium oxide to soil limited the content of cadmium in plants.

The use of zeolites plays a significant role in the deceleration of nitrogen leaching, which results in the appropriate uptake of other macro- and micronutrients by plants from the soil (Leggo 2000). Kos and Leštan (2003) claim that zeolites used as soil amendments can decrease effectively the mobility of trace elements in soil, which makes them less toxic to plants. In the experiment by Sivitskaya and Wyszkowski (2013), zeolite limited the accumulation of copper and nickel in maize. In the current study, a significant effect of zeolite consisting of lower accumulated quantities of trace elements was only confirmed in the case of nickel and copper in oat roots. As reported by Delgado and Gómez (2016), an increase in the soil pH leads to a lower availability of copper, zinc, manganese, and iron to plants. This is also supported by our findings, where the highest decrease in the content of zinc and manganese in grain, straw, and roots, copper in grain, and iron in straw and roots was observed in oat plants growing on soil treated with calcium oxide, which had the strongest impact on the soil pH, raising it to > 7. On the other hand, in the experiment conducted by Sivitskaya and Wyszkowski (2013), the application of calcium oxide to soil generally favoured higher accumulation of trace elements in maize aerial parts.

According to Mascanzoni (1989), the cobalt bioconcentration coefficient, which expressed ratio of cobalt content in plants to its content in soil, ranges from 0.01–0.3. Field studies by Symanowicz et al. (2014) revealed that phosphorus-potassium fertilisation with a dose of P_50_K_150_ applied to soil cropped with *Galega orientalis* Lam. in 2 years led to the achievement of the optimal cobalt bioconcentration coefficient value, which for each of the treatments tested was within 0.12–0.14, and ranged from 0.12 to 0.16 for the research years. As reported by Yoon et al. (2006), a value of the bioconcentration factor > 1 suggests that a given plant is a preferred choice for phytoextraction treatments. In our experiment, the bioconcentration coefficient values were demonstrably less than 1, which proves that the test plant, i.e. oat, should not be recommended for treatments which aim to reduce the content of cobalt in soils contaminated with this element. The content of cobalt in plants is varied. Generally, roots rather than aerial organs are distinguished by much higher accumulation of cobalt (Mermut et al. 1996). Most often, in soils of pH 3.3 and in certain higher plants, the translocation coefficient of this metal reaches much higher values (Mejstřík and Švácha 1988; Palko and Yli-Halla 1988). In an earlier experiment by Kosiorek and Wyszkowski (2016), it was found that the application of cobalt to soil in doses 0–320 mg kg^−1^ of soil depressed the soil reaction from 6.19 (0 mg Co kg^−1^ of soil) to 5.38 (320 mg Co kg^−1^ of soil), which, in line with the above considerations, should lead to higher values of the translocation coefficient for this metal. In this study, under the influence of the increasing cobalt doses added to soil, the cobalt translocation coefficient rose from 0.343 (0 mg Co kg^−1^ of soil) to 0.587 (160 mg Co kg^−1^ of soil), which agrees with the above assumptions. According to Lotfy and Mostafa (2014), the transfer coefficient for cobalt in sunflower plants is higher than in cotton. These authors also showed that the said coefficient is slightly higher in value for plants grown on sandy soils (from 0.08 to 0.18) than on loamy ones (from 0.06 to 0.18).

## Conclusion

In the series without amendments, the increasing doses of cobalt had a significant effect by decreasing the yields of oat grain and straw and the mass of its roots. Also, lower tolerance index values were noted in the objects polluted with cobalt, especially with its highest dose. The application of manure had the strongest effect on increasing the mass of particular organs of the test plant, while the application of charcoal led to a significant decrease in this respect. Application of all substances to soil, and especially manure and calcium oxide, resulted in higher tolerance index Ti values.

The growing contamination of soil with cobalt caused a significant increase in the content of this element in oat (the highest in straw, and the lowest in grain) and in the values of the translocation coefficient, in contrast to the effects noted with respect to the bioconcentration and transfer coefficients. All the substances applied to soil reduced the content of cobalt and its bioconcentration in oat straw, in opposition to grain and roots, limited its translocation, but elevated the transfer of this element from soil to plants.

Soil contamination with cobalt promoted the accumulation of lead and copper in grain, cadmium, lead, nickel, zinc, manganese, and iron in straw, as well as cadmium, nickel, zinc, and manganese in oat roots. As the cobalt dose increased, the content of other trace elements in oat organs either decreased or did not show any unambiguous changes. The impact of all substances applied to soil on the content of trace elements, and especially on the accumulation of zinc, copper, manganese, iron, and lead, was stronger in straw and roots than in oat grain. Most of the soil amendments led to the reduction in the zinc, copper, manganese, and iron content, and increased accumulation of lead in oat straw and roots, compared to the series without soil amendments. The effect of these substances on the content of trace elements in grain and other micronutrients in straw and roots of oat depended on the type of a substance and an organ of oat. Of all the tested substances, the strongest influence on the content of trace elements was produced by calcium oxide in straw and roots and by zeolite in roots, whereas the weakest effect was generated by manure in oat grain.

## References

[CR1] Aery NC, Jagetiya BL (2000). Effect of cobalt treatments on dry matter production of wheat and DTPA extractable cobalt content in soils. Commun Soil Sci Plant Anal.

[CR2] Ali B, Hayat S, Hayat Q, Ahmad A (2010). Cobalt stress affects nitrogen metabolism, photosynthesis and antioxidant system in chickpea (*Cicer arietinum* L.). J Plant Interact.

[CR3] Ali H, Khan E, Sajad MA (2013). Phytoremediation of heavy metals-concepts and applications. Chemosphere.

[CR4] Bhattacharyya P, Chakrabarti K, Chakraborty A, Tripathy S, Kim K, Powell MA (2008). Cobalt and nickel uptake by rice and accumulation in soil amended with municipal solid waste compost. Ecotoxicol Environ Saf.

[CR5] Bibak A (1994). Uptake of cobalt and manganese by winter wheat from a sandy loam soil with and without added farmyard manure and fertilize nitrogen. Commun Soil Sci Plant Anal.

[CR6] Brodowska MS (2003). Effect of liming and sulphur fertilization on the growth and yielding of spring forms of wheat and rape. Part I. Spring wheat. Acta Agrophysica.

[CR7] Chaudhari BH, Parmar JK, Mali RH, Bumbadiya NH (2017). Effect of Co level and FYM on growth and yield of fodder maize. Int J Chem Stud.

[CR8] Ciećko Z, Wyszkowski M, Krajewski W, Zabielska J (2001). Effect of organic matter and liming on the reduction of cadmium uptake from soil by triticale and spring oilseed rape. Sci Total Environ.

[CR9] Delgado A, Gómez JA, Villalobos FJ, Fereres E (2016). The soil physical, chemical and biological properties. Principles of agronomy for sustainable agriculture.

[CR10] Dell Inc (2016) Dell Statistica (data analysis software system), version 13. software.dell.com

[CR11] El-Sheekh MM, El-Naggar AH, Osman MEH, El-Mazaly E (2003). Effect of cobalt on growth, pigments and the photosynthetic electron transport in *Monoraphidium minutum* and *Nitzchia perminuta*. Braz J Plant Physiol.

[CR12] Eprikashvili L, Zautashvili M, Kordzakhia T, Pirtskhalava N, Dzagania M, Rubashvili I, Tsitsishvili V (2016). Intensification of bioproductivity of agricultural cultures by adding natural zeolites and brown coals into soils. Ann Agrar Sci.

[CR13] Finžgar N, Kos B, Leštan D (2006). Bioavailability and mobility of Pb after soil treatment with different remediation methods. Plant Soil Environ.

[CR14] Gad N (2006). Increasing the efficiency of nitrogen fertilization through cobalt application to pea plant. Res J Agric Biol Sci.

[CR15] Gál J, Hursthouse A, Tatner P, Stewart F, Welton R (2008). Cobalt and secondary poisoning in the terrestrial food chain: data review and research gaps to support risk assessment. Environ Int.

[CR16] Garbisu C, Alkorta I (2003). Basic concepts on heavy metal soil bioremediation. Eur J Miner Process Environ Prot.

[CR17] Gujejiani B, Kardava M, Andronikashvili T (2002) Prospects of application of some rocks of Georgian deposits in combination with poultry manure in growing the maize. pp 23

[CR18] Hemantaranjan A, Trivedi AK, Ram M (2000). Effect of foliar applied boron and soil applied iron and sulphur on growth and yield of soybean (*Glycine max* L. Merr.). Indian J Plant Physiol.

[CR19] Homer FA, Morrison RS, Brooks RR, Clemens J, Reeves RD (1991). Comparative studies of nickel, cobalt, and copper uptake by some nickel hyperaccumulators of the genus *Alyssum*. Plant Soil.

[CR20] International Plant Nutrition Institute (2015) Cobalt. Nutri-Facts. Agronomic fact sheets on crop nutrients. North American Edition. http://www.ipni.net/publication/nutrifacts-na.nsf/0/5D2097137F73C07F85257EA8006297B0/$FILE/NutriFacts-NA-15.pdf

[CR21] Ismail AS, Abdel-Sabour MF, Abou-Naga H (1996). Accumulation of heavy metals by plants as affected by application of organic wastes. Egypt J Soil Sci.

[CR22] Jayakumar K, Jaleel CA, Vijayarengan P (2007). Changes in growth, biochemical constituents, and antioxidant potentials in radish (*Raphanus sativus* L.) under cobalt stress. Turk J Biol.

[CR23] Kabata-Pendias A, Mukherjee AB (2007). Trace elements from soil to human.

[CR24] Kalavrouziotis IK, Koukoulakis PH, Manouris G, Papadopoulos AH (2009). Interactions between cadmium, lead, cobalt, and nickel in broccoli, irrigated with treated municipal wastewater. Eur Water.

[CR25] Kandil H, Farid IM, El-Maghraby A (2013). Effect of cobalt level and nitrogen source on quantity and quality of soybean plant. J Basic Appl Sci Res.

[CR26] Kos B, Leštan D (2003). Induced phytoextraction/soil washing of lead using biodegradable chelate and permeable barriers. Environ Sci Technol.

[CR27] Kosiorek M, Wyszkowski M (2016). Selected properties of cobalt-contaminated soil following the application of neutralising substances. Ochr Srod Zas Nat.

[CR28] Kovačević V, Rastija M (2010). Impacts of liming by dolomite on the maize and barley grain yields. Poljoprivreda/Agriculture.

[CR29] Kumpiene J, Lagerkvist A, Maurice C (2008). Stabilization of As, Cr, Cu, Pb and Zn in soil using amendments-a review. Waste Manag.

[CR30] Kuzyakov Y, Subbotina I, Chen H, Bogomolova I, Xu X (2009). Black carbon decomposition and incorporation into soil microbial biomass estimated by ^14^C labeling. Soil Biol Biochem.

[CR31] Leggo PJ (2000). An investigation of plant growth in an organo-zeolitic substrate and its ecological significance. Plant Soil.

[CR32] Liu J, Reid RJ, Smith FA (2000). The mechanism of cobalt toxicity in mung beans. Physiol Plant.

[CR33] Lotfy SM, Mostafa AZ (2014). Phytoremediation of contaminated soil with cobalt and chromium. J Geochem Explor.

[CR34] Mascanzoni D (1989). Long-term transfer from soil to plant of radioactive corrosion products. Environ Pollut.

[CR35] McGrath SP, Chaudri AM, Giller KE (1995). Long-term effects of land application of sewage sludge: soils, microorganisms and plants. J Ind Microbiol.

[CR36] Mejstřík V, Švácha J (1988). Concentrations of Co, Cd, Ni, and Zn in crop plants cultivated in the vicinity of coal-fired power plants. Sci Total Environ.

[CR37] Mermut AR, Jain JC, Song L, Kerrich R, Kozak L, Jana S (1996). Trace element concentrations of selected soils and fertilizers in Saskatchewan, Canada. J Environ Qual.

[CR38] Mleczek M, Gąsecka M, Drzewiecka K, Goliński P, Magdziak Z, Chadzinikolau T (2013). Copper phytoextraction with willow (*Salix viminalis* L.) under various Ca/Mg ratios. Part 1. Copper accumulation and plant morphology changes. Acta Physiol Plant.

[CR39] Mulligan CN, Yong RN, Gibbs GF (2001). Remediation technologies for metal-contaminated soils and groundwater: an evaluation. Eng Geol.

[CR40] Muthaura C, Mucheru-Muna M, Zingore S, Kihara J, Muthamia J (2017). Effect of application of different nutrients on growth and yield parameters of maize (*Zea mays*), case of Kandara Murang’a County. ARPN J Agric Biol Sci.

[CR41] Ostrowska A, Gawliński S, Szczubiałka Z (1991). Methods for analysis and evaluation of soil and plant properties.

[CR42] Palit S, Sharma A, Talukder G (1994). Effects of cobalt on plants. Bot Rev.

[CR43] Palko J, Yli-Halla M (1988). Solubility of Co, Ni, and Mn in some extractants in a Finnish acid sulphate soil area. Acta Agric Scand.

[CR44] Rinkins GA, Hollendorf VF (1982) A balanced diet of plant macro-and micronutrients. Zinatne, Riga

[CR45] Sillanpää M, Jansson H (1992) Status of cadmium, lead, cobalt and selenium in soils and plants of thirty countries. FAO Soils Bulletin 65, Food & Agriculture Org, Rome, p 195

[CR46] Sivitskaya V, Wyszkowski M (2013). Effect of heating oil and neutralizing substances on the content of some trace elements in maize (*Zea mays* L.). Ecol Chem Eng A.

[CR47] Stefanescu M (2004). Role of manure in increasing soil fertility and yield of wheat and maize. Rom Agric Res.

[CR48] Symanowicz B, Kalembasa S, Malinowska E, Wysokiński A (2014). Changes in the content of zinc and cobalt in plants and soil caused by absorption by goat’s rue (*Galega orientalis* Lam.) biomass and the bioaccumulation factors under the influence of fertilization with phosphorus and potassium. J Elem.

[CR49] Van Zwieten L, Kimber S, Morris S, Chan KY, Downie A, Rust J, Joseph S, Cowie A (2010). Effects of biochar from slow pyrolysis of papermill waste on agronomic performance and soil fertility. Plant Soil.

[CR50] Wyszkowski M (2017). Effect of contamination with copper and mineral or organic amendments on the content of trace elements in soil. Environ Prot Eng.

[CR51] Wyszkowski M, Sivitskaya V (2013). Effect of heating oil and neutralizing substances on the content of some trace elements in soil. Fresenius Environ Bull.

[CR52] Wyszkowski M, Ziółkowska A (2009). Role of compost, bentonite and calcium oxide in restricting the effect of soil contamination with petrol and diesel oil on plants. Chemosphere.

[CR53] Wyszkowski M, Ziółkowska A (2011). The importance of relieving substances in restricting the effect of soil contamination with oil derivatives on plants. Fresenius Environ Bull.

[CR54] Yadov DV, Khanna SS (2002). Role of cobalt on nitrogen fixation-a review. Agric Rev.

[CR55] Yoon J, Cao X, Zhou Q, Ma LQ (2006). Accumulation of Pb, Cu, and Zn in native plants growing on a contaminated Florida site. Sci Total Environ.

[CR56] Zaborowska M, Kucharski J, Wyszkowska J (2016). Biological activity of soil contaminated with cobalt, tin and molybdenum. Environ Monit Assess.

[CR57] Zeid IM (2001). Responses of *Phaseolus vulgaris* to chromium and cobalt treatment. Biol Plant.

[CR58] Zengin FK, Munzuroglu O (2005). Effects of some heavy metals on content of chlorophyll, proline and some antioxidant chemicals in bean (*Phaseolus vulgaris* L.) seedlings. Acta Biol Cracov Ser Bot.

